# Understanding cost variation in medical and health sciences education: An institutional perspective

**DOI:** 10.1016/j.hpopen.2026.100165

**Published:** 2026-02-18

**Authors:** Ahmad Reshad Osmani

**Affiliations:** Assistant Professor of Economics, Department of Economics and Finance, Louisiana State University Shreveport One University Place, Shreveport 71115, LA, USA

**Keywords:** Medical education, Cost analysis, Fragile states, Gender disparities, Afghanistan

## Abstract

Strengthening the health workforce is a central policy challenge in fragile and low-income settings, yet governments often lack basic information on what it costs to train health professionals. This study provides the first institutional level evidence on the structure and magnitude of medical education costs in Afghanistan using detailed administrative and financial records from Kabul Medical University. A retrospective costing approach combined with a step-down cost accounting framework is used to allocate all direct and shared expenditures across faculties and across years of study. The results reveal large differences in per student costs across programs, with Curative Medicine requiring substantially more resources than Stomatology, Nursing, and Public Health. Support services account for a large share of total expenditure, and resource use rises sharply in clinically intensive years. A sensitivity analysis that varies personnel costs, allocation rules, and price adjustments shows that these patterns remain stable. The findings indicate that fiscal pressures, gender imbalances in enrollment, and inefficient allocation of support costs limit the effectiveness of current training investments. The study provides a quantitative foundation for policies that seek to improve the financial sustainability of medical universities, align training capacity with national health needs, and strengthen resource tracking systems in fragile and low-income contexts.

## Highlights

• Analyzes the cost of medical education in post-conflict Afghanistan.
• Uses detailed 2019–2020 expenditure data from Kabul Medical University.
• Finds large cost variation across faculties, highest in clinical programs.
• Identifies major budget share in administrative and support services.
• Highlights gender disparities in high-cost programs affecting healthcare.

## 1 Introduction

Higher education is widely recognized as a foundational driver of human capital formation, economic growth, and institutional development. Its role is especially significant in social sectors that influence public welfare, such as healthcare. Within this field, medical and health sciences education holds a central position. It prepares the physicians, nurses, midwives, dentists, public health experts, and other providers who are essential for expanding service coverage, improving health outcomes, and strengthening the resilience of health systems. In low income and post conflict settings, where health systems often face severe shortages of workers and limited resources, the ability to train and retain qualified health professionals is critical for both immediate service delivery and long-term system stability.

Although decades of empirical work have documented substantial private and social returns to schooling [1], [2], the cost side of higher education remains comparatively understudied. Much of the existing research focuses on primary and secondary schooling, and far less is known about cost structures at the tertiary level. Medical education presents even greater analytic challenges because it requires specialized faculty, laboratory facilities, clinical training sites, and programs that last many years. These characteristics make medical training far more resource intensive than most other academic fields. Yet detailed institutional level cost analyses remain rare in low- and middle-income countries and are almost entirely absent in places that have experienced conflict or prolonged fragility. Reliable cost data are essential for enrollment planning, budget allocation, resource prioritization, and for ensuring that training systems align with national health needs.

This gap is especially important because medical education often absorbs a large share of public education budgets relative to the number of students it serves. Facilities for clinical instruction, simulation laboratories, and specialized departments require considerable financial investment. Without precise cost information, governments and universities may struggle to evaluate efficiency, identify cross faculty disparities, or assess whether current allocations are equitable across programs and gender groups. The absence of systematic cost tracking can also limit the effectiveness of performance-based budgeting and can hinder long term planning for infrastructure investment and clinical training capacity.

These considerations are especially relevant in Afghanistan. The country’s higher education system has been shaped by many years of conflict, political instability, and uneven institutional support. Although the period after 2001 brought improvements in educational access and health service coverage, Afghanistan continues to face an acute shortage of qualified health professionals. National densities of physicians, nurses, and midwives remain far below global benchmarks, and shortages are most severe in rural and underserved communities. Sociocultural norms also restrict the ability of many women to seek care from male providers, which increases the need for trained female physicians and nurses. These challenges highlight the importance of strong and efficient medical training institutions.

Public medical universities serve as the primary pathway for training Afghanistan’s health workforce. Kabul Medical University is the oldest and most prominent among them. Established in 1932, the university has trained many generations of physicians, nurses, dentists, and public health professionals who work across the country. KMU offers programs in Curative Medicine, Nursing, Public Health, and Stomatology. Each program differs in length and structure, and each has unique requirements for laboratory work, clinical rotations, and instructional staffing. Despite its central position in the national health education system, KMU has never been the subject of a comprehensive cost analysis. Financial records are maintained through paper-based systems, cost centers are not systematically tracked, and program level budgeting is not used. These limitations make it difficult to assess efficiency, understand resource allocation patterns, or plan for expansion of training capacity.

International literature underscores the urgent need for more evidence in this area. Training health professionals in resource constrained settings requires careful financial planning to ensure sustainability and alignment with health system needs [3], [4]). Studies also show that higher densities of skilled health workers are strongly associated with improvements in maternal, infant, under five, and neonatal mortality [5]. Despite this, many medical education systems in developing countries operate with limited cost transparency and weak financial monitoring, which complicates reform efforts [6]. These challenges have encouraged researchers and institutions to explore structured costing approaches that are suitable for data limited environments. Step down cost accounting is one such approach. It has been widely used in hospital finance to allocate both direct and shared costs across departments and service units in a transparent and systematic manner [7], [8]. Although it has not been used extensively in higher education, it offers a practical method for estimating program level costs in complex institutions such as medical universities.

This study responds to these gaps by presenting the first detailed cost analysis of medical education in a post conflict setting. Using administrative, academic, and expenditure data from Kabul Medical University for the 2019 to 2020 academic year, the study applies a step-down cost accounting framework to estimate total and per student costs across faculties and years of study. The method distinguishes between service cost centers, such as teaching departments and clinical training units, and support cost centers, such as administration, library services, maintenance units, student dormitories, and donor supported faculty development programs. By allocating costs across these functional areas, the study produces a detailed picture of how resources are used within the university and how cost structures differ by program and stage of training.

The objective of this analysis is to generate rigorous and policy relevant cost estimates that can guide budget planning, resource allocation, donor coordination, and workforce development strategies in Afghanistan’s health education sector. The findings contribute to the broader literature on education finance, health workforce planning, and cost accounting in fragile contexts. They also offer insights for other countries that rely on publicly funded medical universities but lack reliable cost information for decision making. By demonstrating the feasibility of applying a structured costing method in a post conflict environment, the study provides a model that can be adapted by similar institutions in low income and fragile settings.

## 2 Materials and Methods

### 2.1 Data

This study is based on detailed administrative and financial records from (KMU, the primary public institution responsible for training medical professionals in Afghanistan. The analysis uses a single retrospective costing year covering the academic period from March 2019 to March 2020. This period was selected because it corresponds to the most complete and verifiable set of institutional records available prior to subsequent structural and political disruptions. The dataset includes information on personnel expenditures, operational costs, academic staffing, infrastructure usage, student enrollment, and donor-supported faculty development activities. Data were collected through direct, in-person collaboration with the university’s finance office, academic affairs division, human resources department, and registrar’s office. More than one hundred institutional documents, including payroll records, budget statements, procurement logs, facility maintenance reports, and enrollment registries, were reviewed, transcribed, and cross-validated through repeated consultations with senior administrators, department heads, and faculty deans.

KMU delivers education through four academic faculties: Curative Medicine, Nursing, Public Health, and Stomatology. Program duration varies across faculties, with Curative Medicine structured over seven academic years, Stomatology over six years, and Nursing and Public Health over four years each. During the study period, total enrollment was 1,764 students, comprising 743 in Curative Medicine, 360 in Nursing, 250 in Public Health, and 411 in Stomatology. Enrollment records were disaggregated by program, year of study, and gender. Gender disparities were particularly pronounced in Curative Medicine, where female students constituted only 27 percent of enrollment, whereas women represented more than half of students in the other faculties. All financial data were originally recorded in Afghani and subsequently converted to United States dollars using the official average exchange rate for the study period, which was 78.8 Afghani per United States dollar. Because the cost covers a single academic year, no intra-period inflation adjustment was required. However, for comparability with external literature and international benchmarks, results are reported in constant 2019 United States dollars. This standardization was carried out by adjusting nominal expenditures to 2019 values using the appropriate consumer price index factor and then converting the resulting amounts into United States dollars using the average exchange rate for the study period.

When specific cost items were missing or inconsistently documented, proportional allocation procedures were applied. Administrative costs were allocated according to staff size and payroll share, while facility-related costs were allocated based on usable floor space and occupancy ratios. These allocation rules were developed in consultation with university officials to ensure that they reflected institutional realities and local practice. Expenditures incurred by external clinical training sites, such as hospitals where students complete rotations, were not included. Nevertheless, all internal costs directly associated with the delivery of medical education were captured, providing a comprehensive institutional perspective on resource use in a fragile, low-income setting.

### 2.2 Methods

This study adopts a retrospective institutional costing approach to estimate the unit cost of medical education at KMU. Within this broader costing framework, step-down cost accounting (SDCA) is used as the primary resource allocation technique. That is, SDCA is treated not as the overall costing method itself, but as a structured procedure for redistributing shared and indirect (overhead) costs from support departments to final service units. The overall approach therefore consists of retrospective costing combined with step-down allocation to derive the full economic cost attributable to each academic faculty. Resources were identified, measured, and valued using an ingredient-based strategy. First, physical and human resources were identified from institutional records and key informant interviews, including teaching and non-teaching staff, buildings and classrooms, laboratories, utilities, and recurrent inputs. Second, quantities of these inputs were measured using official documentation such as payroll lists, timetables, space inventories, utility bills, and procurement records. Third, resources were valued using actual expenditure data (for example, salaries, allowances, procurement prices, and maintenance contracts). For resources shared across multiple departments, proportional allocation keys were defined to reflect observed patterns of use.

All university departments were classified as either support cost centers or service cost centers. Support cost centers include central administration, finance, human resources, maintenance, dormitories, food services, and library and information technology. Service cost centers comprised the four academic faculties that deliver formal instruction. The step-down procedure followed a sequential logic, whereby costs from each support center were allocated to remaining support and service centers according to predefined allocation criteria, after which that center was “closed” and excluded from subsequent rounds of allocation.

Let i index departments and let $C_{i}$ represent the direct cost incurred by department i. For each support unit, costs were distributed to other departments using allocation weights $\alpha_{\mathrm{ij}}$, where $\alpha_{\mathrm{ij}}$ denotes the proportion of support unit i’s cost allocated to department j, and:

(1) $$\sum_{j}\alpha_{\mathrm{ij}}=1.$$

After all support units had been fully allocated, the total cost of each academic faculty j was given by:

(2) $$\begin{array}{c} TC_{j}=DC_{j}+\sum_{i\in S}\alpha_{\mathrm{ij}}\;FC_{i}, \end{array}$$

where $TC_{j}$ is the total cost of faculty j, $DC_{j}$ is its direct cost, S denotes the set of support units, and $FC_{i}$ is the fully allocated cost of support unit i after all redistribution steps.

The average per-student cost for each faculty was then calculated as:

(3) $$\begin{array}{c} \mathrm{PS}C_{j}=\frac{TC_{j}}{N_{j}}, \end{array}$$

where $N_{j}$ is the number of students enrolled in faculty j. This yields a faculty-level unit cost of medical education.

To account for heterogeneity in instructional intensity across different years of study, we further disaggregated costs using year-specific intensity weights. These weights were constructed using a composite index that combined information on curriculum complexity, number of contact teaching hours, laboratory and clinical requirements, and faculty time allocation. Data for this index were derived from official curriculum plans, teaching schedules, and structured interviews with senior academic staff. While the use of intensity weights is consistent with approaches seen in the education costing literature, the specific weighting scheme applied here was developed by the authors to reflect the institutional structure and pedagogical design of KMU.

Let $\omega_{\mathrm{jt}}$ denote the instructional intensity weight for year t in faculty j, and let $Y_{\mathrm{jt}}$ represent the number of students enrolled in that year. The year-specific per-student cost was then defined as:

(4) $$\begin{array}{c} \mathrm{PS}C_{\mathrm{jt}}=\frac{\omega_{\mathrm{jt}}\;TC_{j}}{\sum_{t}\omega_{\mathrm{jt}}\;Y_{\mathrm{jt}}}, \end{array}$$

which assigns higher cost shares to years with greater instructional and resource demands, such as those involving clinical rotations or extensive laboratory work. This framework allows us to distinguish between preclinical and clinical years and to examine how costs evolve across the educational trajectory.

A sensitivity analysis was conducted to assess how changes in key assumptions influence the estimated per student costs. Two scenarios were examined. The first scenario removed the twenty percent inflation adjustment applied in the baseline estimates and recalculated all per student costs using nominal values from the study year. This scenario tested whether restating expenditures to the original price level altered the relative ranking of faculty costs. The second scenario varied faculty enrollment by ten percent above and below observed levels while holding total faculty cost constant. This scenario assessed how fluctuations in enrollment, a major determinant of per student cost, affected the distribution of costs across programs. These two scenarios were chosen because the necessary information is available in the dataset and they reflect realistic changes that institutions may experience from year to year.

## 3 Results

### 3.1 Total and faculty-level cost estimates

Table 1 reports the total and per student costs for the four academic faculties at Kabul Medical University during the 2019 to 2020 academic year. Total institutional expenditure was 4.12 million United States dollars after adjusting for the wage and price level of the following fiscal year. Curative Medicine recorded a total cost of 2.2 million dollars and a per student cost of 22,171 dollars. Stomatology recorded a per student cost of 7,867 dollars. Nursing recorded a per student cost of 3,423 dollars, and Public Health recorded a per student cost of 1,439 dollars.

**Table 1:** Total and per-student adjusted costs by faculty, KMU, 2019–2020.

Faculty	Total Cost (USD)	Students	Per Student Cost (USD)
Curative Medicine	2,200,000	743	22,171
Stomatology	840,000	411	7,867
Nursing	1,232,400	360	3,423
Public Health	359,750	250	1,439

**Fig. 1:**
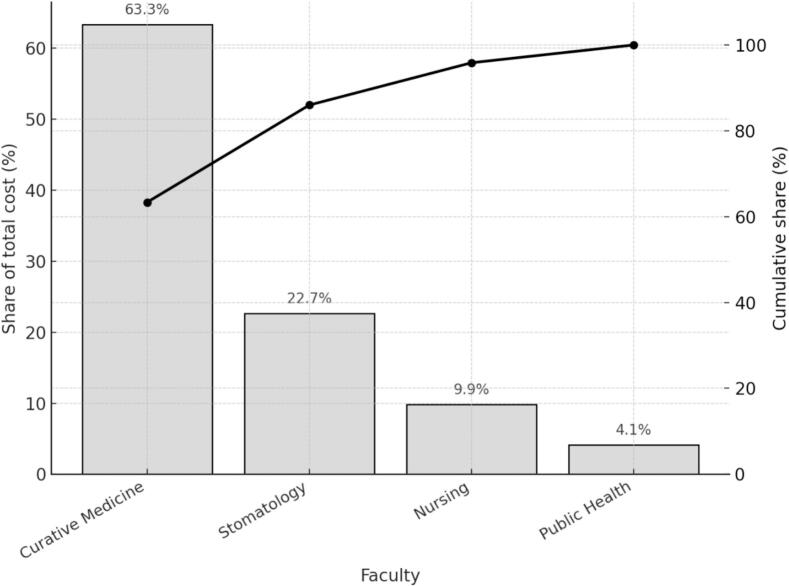
Pareto distribution of total adjusted costs by faculty, KMU, 2019–2020.

Fig. 1 displays the distribution of total adjusted costs across the four academic faculties. The figure shows each faculty’s share of total expenditure and the cumulative share of institutional spending. Curative Medicine accounts for slightly more than half of total costs. Stomatology represents the next largest share, followed by Nursing and Public Health. The cumulative distribution indicates that Curative Medicine and Stomatology together account for more than seventy percent of all institutional spending.

### 3.2 Support cost allocation and institutional spending patterns

Table 2 presents the distribution of support costs by functional area. Support units accounted for 46 percent of total institutional expenditure. The administrative office accounted for 11 percent of total costs. Dormitory and food services accounted for 9 percent. Donor supported development programs accounted for 12 percent. Facility maintenance accounted for 10 percent. Library and information technology services accounted for 4 percent.

**Table 2:** Distribution of support costs.

Support Cost Center	Percentage of Total Cost (%)	Cost in USD
Administration	11	453,200
Dormitories and Food Services	9	370,800
Library and IT	4	164,800
Donor-Funded Development	12	494,400
Facility Maintenance	10	412,000

**Fig. 2:**
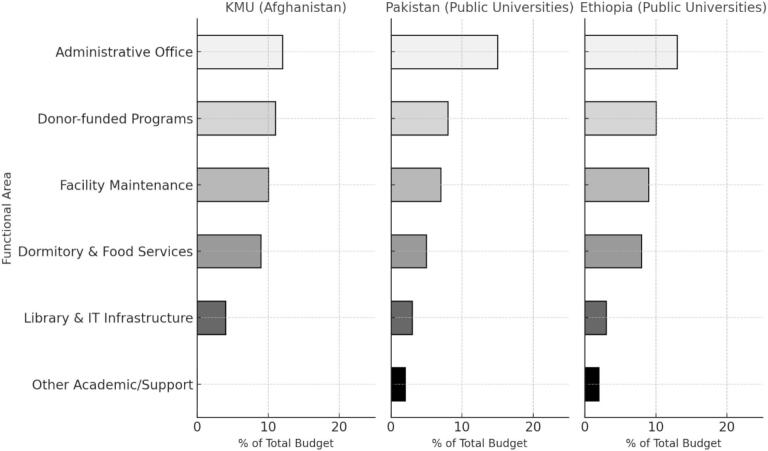
Budget allocation by functional area in KMU (Afghanistan), Pakistan, and Ethiopia.Data for Pakistan and Ethiopia are based on published estimates from public university expenditure studies [[9], [10]].

Fig. 2 shows the distribution of university expenditures by functional area. The figure displays the share of total spending accounted for by administration, dormitory and food services, library and information technology, donor supported development programs, and facility maintenance. Each category is presented as a proportion of the total institutional budget for the 2019 to 2020 academic year.

### 3.3 Costs across years of study in Curative Medicine

Table 3 reports per student costs for each of the seven years of the Curative Medicine program. The cost for Year 1 is one thousand eight hundred dollars per student. The cost for Year 2 is one thousand nine hundred eighty dollars. The cost for Year 3 is two thousand one hundred sixty dollars. The cost for Year 4 is two thousand three hundred forty dollars. The cost for Year 5 is two thousand eight hundred eighty dollars. The cost for Year 6 is three thousand two hundred forty dollars. The cost for Year 7 is three thousand six hundred dollars.

**Table 3:** Yearly cost distribution in Curative Medicine.

Year of Study	Weight (Instructional Intensity)	Per Student Cost (USD)
Year 1	1.0	1800
Year 2	1.1	1980
Year 3	1.2	2160
Year 4	1.3	2340
Year 5	1.6	2880
Year 6	1.8	3240
Year 7	2.0	3600

Instructional intensity weights are assigned based on clinical complexity, staff involvement, and equipment needs.

**Fig. 3:**
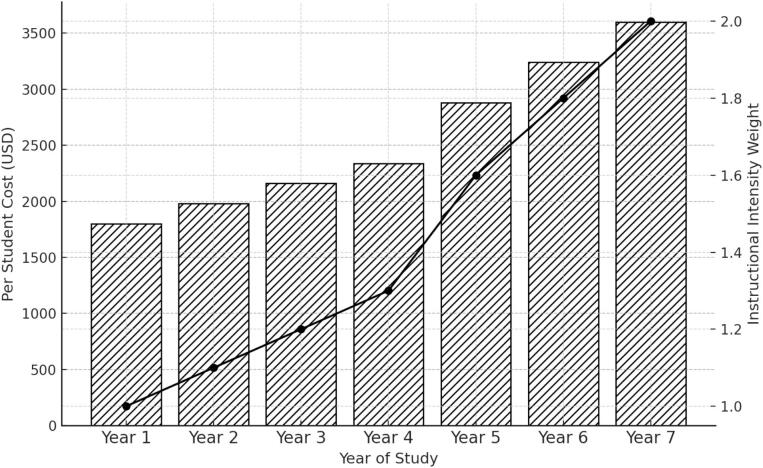
Per-student cost and instructional intensity by year of study in the Curative Medicine program at KMU.

Fig. 3 displays per student costs for each year of the Curative Medicine program alongside the instructional intensity weights assigned to each year. The figure shows the numerical relationship between annual costs and the corresponding weights for all seven years of study.

### 3.4 Faculty-to-student ratios and labor utilization

Table 4 reports faculty to student ratios for the four academic faculties. Curative Medicine has a ratio of one faculty member for every five students. Stomatology has a ratio of one to seven. Nursing has a ratio of one to nine. Public Health has a ratio of one to ten.

**Table 4:** Faculty-to-student ratios by Faculty.

Faculty	Total Faculty	Total Students	Faculty-to-Student Ratio
Curative Medicine	150	743	1:5
Stomatology	60	411	1:7
Nursing	40	360	1:9
Public Health	25	250	1:10

Ratios reflect average teaching loads for full-time faculty during the 2019–2020 academic year.

### 3.5 Gender disparities and resource equity

Table 5 presents the gender composition and per-student costs across different faculties.

**Table 5:** Gender composition and per-student cost by faculty.

Faculty	Female Share (%)	Male Share (%)	Per-Student Cost (USD)	Resource Intensity*
Curative Medicine	27	73	22,171	High
Stomatology			7867	Medium
Nursing	>50	<50	3423	Low
Public Health	>50	<50	1439	Low

This disparity has both ethical and practical implications. In rural Afghanistan, where cultural norms limit access to male healthcare providers, the underrepresentation of women in medical faculties can hinder healthcare delivery and worsen maternal health outcomes. In Bangladesh, although a growing proportion of medical graduates are female, with female physicians slightly outnumbering males (52% vs. 48%); the persistence of male-dominated academic and professional environments continues to shape service accessibility (Hossain, 2019). In Sudan, women's lower secondary and tertiary enrollment rates—only around 41% of secondary students are female, suggest broader structural barriers that likely extend to medical training programs and health service delivery [11]. Addressing this imbalance at KMU requires not only inclusive admissions policies but also targeted scholarships and support mechanisms to expand female participation in high-cost medical programs.

### 3.6 Sensitivity analysis

Table 6 presents the results of the sensitivity analysis combining inflation adjustment and enrollment variation scenarios. Removing the inflation adjustment reduces all per student costs by sixteen-point seven percent. Increasing enrollment by ten percent lowers per student costs, while decreasing enrollment raises them proportionally. The relative ranking of programs remains unchanged under all scenarios.

**Table 6:** Combined sensitivity analysis of Per Student cost estimates.

Faculty	Baseline adjusted cost (USD)	Unadjusted cost (USD)	Enrollment + 10 percent (USD)	Enrollment − 10 percent (USD)
Curative Medicine	22,171	18,476	20,155	24,635
Stomatology	7,867	6,556	7,152	8,741
Nursing	3,423	2,853	3,112	3,803
Public Health	1,439	1,199	1,308	1,599

Unadjusted costs remove the twenty percent inflation adjustment applied in baseline estimates. Enrollment variation scenarios increase or decrease the number of enrolled students by ten percent while holding total faculty cost constant.

## 4 Discussion

The cost estimates from this study show clear differences in the resource requirements of the four academic faculties at KMU. Curative Medicine and Stomatology exhibit substantially higher per student expenditures than Nursing and Public Health, reflecting the longer duration of clinical programs, their intensive instructional demands, and their reliance on specialized facilities. These patterns are consistent with evidence from other low- and middle-income contexts where medical and dental training requires greater investment than non-clinical programs [[12], [13], [14]]. The observed cost structure at KMU therefore aligns with broader empirical findings on the nature of medical education in resource constrained settings.

Support services account for almost half of total institutional expenditure. Administrative operations, dormitories, food services, library and information technology services, and donor supported development programs make up the majority of these support costs. Similar distributions appear in Pakistan and Ethiopia, where administrative and other non-instructional activities account for a significant share of total spending [[9], [10]], and in the United States, where non instructional expenditures represent an important component of institutional budgets [15]. The patterns observed at KMU are therefore consistent with cost structures documented in universities that operate with centralized services and shared resource environments.

Per student costs in Curative Medicine at KMU increase steadily across the later years of study. This corresponds with the transition from foundational coursework to more intensive clinical exposure. Evidence from Malawi and Tanzania shows similar cost escalation in clinical phases of training, where supervision, rotation schedules, and facility use generate substantial resource demands [16], [17]. The year level cost pattern at KMU therefore mirrors established cost trajectories found in other medical training systems in low-income settings.

Differences in faculty to student ratios across programs at KMU provide additional insight into the distribution of instructional resources. Curative Medicine and Stomatology maintain lower ratios than Nursing and Public Health. This aligns with evidence from other studies indicating that instructional labor, especially faculty time and compensation, is a central driver of cost variation across academic programs [18], [19]. Although salary structures at KMU differ from those in higher income systems, the relative differences in staffing intensity follow a well-documented pattern in medical and health sciences education.

The gender composition of KMU students also offers important context for interpreting the cost structure. Women represent a minority in Curative Medicine, the most resource intensive faculty, while they constitute the majority in Nursing, Public Health, and Stomatology. Similar patterns of gender-based distribution appear in regional and global studies. In Bangladesh, women now make up a slightly higher proportion of physicians, yet gender norms continue to influence training and career progression [20]. In Sudan, lower female participation in secondary and tertiary education reflects structural challenges that limit women’s entry into medical programs [11]. International evidence also highlights persistent gender inequalities in health worker training and employment [21]. The gender distribution at KMU therefore fits within broader global patterns linking gender, educational access, and workforce composition.

The findings support several institution level recommendations that align directly with the empirical results. First, the concentration of support costs at KMU demonstrates the usefulness of adopting a structured cost accounting system. Step down cost accounting is particularly appropriate for KMU because it accommodates paper based financial systems, pooled administrative structures, and centralized budgeting. The method allows consistent allocation of support costs and generates cost information that can be updated regularly for annual budgeting and planning. Second, while donor supported expenditures are included within support costs and are fully allocated across faculties through the step-down process, the results underscore the importance of improved tracking of donor inputs before they are incorporated into institutional budgets. This would strengthen financial transparency and enhance KMU’s ability to assess the contribution of external support.

Third, the evidence supports consideration of differentiated funding across faculties at KMU. The instructional intensity weights used in the study provide a structured representation of relative demand across years and programs. These weighted cost patterns offer a justification for faculty level budgets that consider the variation in instructional and clinical training requirements. Because the weighting method is based on curriculum structure and documented instructional loads, it provides a transparent basis for linking funding levels to program needs.

Finally, the gender distribution at KMU indicates that resource planning would benefit from initiatives that expand female participation in programs with the highest resource requirements. While the cost analysis does not identify the causes of gender patterns, the results clearly show the intersection between gender composition and the distribution of public expenditure across programs.

The combined sensitivity analysis shows that the main cost patterns are stable across alternative assumptions. Removing the inflation adjustment reduces all per student costs by a uniform proportion, and varying enrollment produces predictable changes in cost magnitude. In all cases, Curative Medicine remains the most resource intensive program, followed by Stomatology, Nursing, and Public Health. The consistency of these rankings indicates that the observed cost differences reflect structural characteristics of instructional requirements rather than artifacts of inflation assumptions or enrollment levels.

## 5 Conclusion

This study provides the first comprehensive examination of the cost structure of medical and health sciences education at KMU using a step-down cost accounting approach. The findings reveal substantial variation in per student expenditure across faculties, with the highest costs concentrated in the clinical programs. Support services absorb a large share of total institutional spending, and costs rise appreciably as students’ progress into the clinical stages of Curative Medicine. Variation in faculty to student ratios and gender composition further illustrates how institutional characteristics influence the distribution of educational resources at KMU.

Several policy implications follow from these results. The clear differences in cost levels across programs highlight the importance of faculty specific budgeting that reflects program duration, instructional intensity, and clinical training requirements. The large share of resources devoted to support functions points to the value of strengthening financial management systems that can track both direct and indirect expenditures on a routine basis. The suitability of step-down cost accounting for KMU lies in its ability to operate effectively in an environment that relies on paper based financial systems and centralized administrative structures. Institutionalizing this approach would enable KMU to generate regular, program level cost information that can inform planning, negotiation with government authorities, and coordination with external partners. In addition, the gender distribution of students suggests that expanding opportunities for women in the more resource intensive programs may be important for future health workforce composition and planning.

This study has several limitations. It analyses data from only one academic year, which may not capture changes in expenditure over time. Costs incurred by external clinical training sites could not be included due to data constraints, resulting in conservative estimates of total training cost. The instructional intensity weights used to distribute faculty level costs across years of study were informed by curriculum review and expert consultation, but they remain approximations of relative resource use. These limitations reflect the available data rather than limitations of the costing framework.

Despite these constraints, the study provides detailed and previously unavailable evidence on the cost of medical and health sciences education at KMU. The findings establish a foundation for more systematic cost monitoring and offer a reference point for institutions operating under similar conditions. The results also contribute to broader efforts to understand the financial requirements of health workforce development in fragile and low-income settings.

## 6 Data availability Statement

The data used in this study can be received upon request. The author signed a contract not to share data without prior approval from KMU administrative offices.

## CRediT authorship contribution statement

**Ahmad Reshad Osmani:** Writing – review & editing, Writing – original draft, Visualization, Validation, Supervision, Software, Resources, Project administration, Methodology, Investigation, Formal analysis, Data curation, Conceptualization.

## Declaration of competing interest

The author declare that they have no known competing financial interests or personal relationships that could have appeared to influence the work reported in this paper.
